# The Prophylactic Effect of Dexmedetomidine 0.008% Versus Brimonidine 0.2% on IOP Elevation After Nd: YAG Laser Capsulotomy

**DOI:** 10.1002/prp2.70227

**Published:** 2026-05-26

**Authors:** Parisa Zakeri, Ghodrat Akhavanakbari, Habib Ojaghi, Firouz Amani

**Affiliations:** ^1^ Ardabil University of Medical Sciences Ardabil Iran; ^2^ Department of Anesthesiology Ardabil University of Medical Sciences Ardabil Iran; ^3^ Department of Ophthalmology Ardabil University of Medical Sciences Ardabil Iran; ^4^ Department of Community Medicine and Biostatistics Ardabil University of Medical Sciences Ardabil Iran

**Keywords:** brimonidine, dexmedetomidine drops, IOP, Nd:YAG laser, posterior capsulotomy, topical

## Abstract

Posterior capsule opacification (PCO) is a common post‐cataract surgery complication treated with Nd:YAG laser posterior capsulotomy, which may cause intraocular pressure (IOP) spikes and threaten vision. Brimonidine and apraclonidine are widely used to prevent such elevations. This prospective, double‐masked, randomized clinical trial evaluated the efficacy of topical dexmedetomidine, a novel ophthalmic drop, in preventing IOP rise after Nd:YAG laser treatment. A total of 111 eyes from 89 pseudophakic patients were randomized to receive dexmedetomidine 0.008% or brimonidine 0.2% one h before the procedure. Patients with glaucoma, baseline IOP > 24 mmHg, keratoconus, corneal edema, prior refractive/corneal surgery, or unstable cardiovascular disease were excluded. IOP was measured with air‐puff tonometry at baseline, 30 min, 4 h, and 24 h post‐laser. Baseline characteristics were comparable. In the dexmedetomidine group, mean IOP values were 16.3 ± 3.6, 14.8 ± 4.7, 17.1 ± 6.2, and 16.7 ± 4.5 mmHg, while in the brimonidine group, they were 16.7 ± 2.9, 13.3 ± 3.9, 13.2 ± 5.5, and 14.2 ± 3.9 mmHg, respectively. At 30 min, brimonidine significantly reduced IOP (*p* = 0.000), whereas dexmedetomidine did not (*p* = 0.116). At 4 and 24 h, IOP increased above baseline with dexmedetomidine but decreased with brimonidine (*p* = 0.001 and *p* = 0.004). Dexmedetomidine was associated with more IOP spikes > 10 mmHg (9% vs. 2%, *p* = 0.035) and IOP > 30 mmHg (7% vs. 2%, *p* = 0.09). No systemic or ocular side effects occurred. Although dexmedetomidine prevented acute IOP surges, its efficacy was inferior to brimonidine. Further studies should explore optimal dosing, formulations, and long‐term safety to clarify its prophylactic potential.

## 1 Introduction

Cataract surgery using phacoemulsification with intraocular lens (IOL) implantation is a frequently performed procedures globally [1, 2]. One of the most common delayed complications of this surgery is posterior capsule opacification (PCO), which affects 20%–40% of patients within 2–5 years and can lead to visual disturbances [1, 3] Nd:YAG laser posterior capsulotomy is widely considered to be the standard treatment of PCO. However, a transient rise in intraocular pressure (IOP) is a frequently reported complication, occurring in 15%–36% of untreated patients [1, 3]. Since IOP spikes are unpredictable and may cause serious conditions such as ischemic optic neuropathy in susceptible individuals, preventive measures are routinely recommended before the procedure.

Topical α2‐adrenergic agonists, such as apraclonidine 0.5% and brimonidine 0.2%, are widely used to prevent IOP spikes [1, 3]. Dexmedetomidine, another selective α2‐adrenergic agonist, has a significantly higher affinity for α2 receptors compared with α1 receptors. due to its sedative and analgesic effects, dexmedetomidine is primarily used as an anesthetic agent without compromising respiratory function [2, 4]. Activation of α2 receptors reduces IOP by mitigating the production of aqueous humor and promoting uveoscleral outflow, effects observed with intravenous dexmedetomidine administration [4, 5, 6, 7]. Additionally, α2 receptor activation induces vasoconstriction, potentially reducing hyperemia, a well‐documented adverse event associated with glaucoma treatments. However, systemic adverse events including bradycardia, hypotension, hypertension, and nausea may limit its use in outpatient settings [2].

Studies have shown that dexmedetomidine, when added to peribulbar blocks during cataract and glaucoma surgeries, reduces IOP similarly to intravenous administration [8, 9, 10, 11]. Recently, topical dexmedetomidine 0.0055% was reported to reduce IOP by 20%–44% in healthy subjects [12], with further studies confirming its efficacy in lowering IOP [4]. To date, no studies have investigated the effectiveness of topical dexmedetomidine in preventing IOP spikes after Nd:YAG laser capsulotomy. The present double‐masked, randomized controlled trial (RCT) aims to assess the prophylactic effects of topical dexmedetomidine 0.008% and brimonidine 0.2% on post‐laser IOP elevation. Additionally, the study will evaluate and compare potential complications of these two medications. This research represents the first investigation into this specific application of dexmedetomidine.

## 2 Materials and Methods

The present study was a double‐masked, randomized controlled trial conducted at the Noor Ophthalmology Clinic, a private practice in Ardabil, Iran, between April 2024 and September 2024. The study included 111 eyes from 89 pseudophakic patients who had undergone uncomplicated cataract surgery and developed blurred vision due to moderate or severe posterior capsule opacification (PCO). Moderate PCO was defined as localized or diffuse opacification of the posterior capsule with poor red reflex, while severe PCO was characterized by very poor red reflex visible on slit‐lamp biomicroscopy, both resulting in measurable decline in best‐corrected visual acuity (BCVA).

Positive history of glaucoma, baseline intraocular pressure (IOP) > 24 mmHg, keratoconus, corneal edema, prior refractive or corneal surgery, or unstable cardiovascular conditions including systolic blood pressure (SBP) > 180 mmHg, diastolic blood pressure (DBP) > 105 mmHg, heart rate < 50 or > 120 bpm, were considered as exclusion criteria. All patients were recruited to the study once they provided written informed consent, and the study protocol was approved by the Ardabil University of Medical Sciences Ethics Committee (Approval No: IR.ARUMS.REC.1402.148; IRCT No: 20231008059648 N1), adhering to the Declaration of Helsinki.

### 2.1 Sample Size Calculation

Using an 85% power, an estimated mean IOP difference of 1.85 mmHg, and standard deviations of 2.2 mmHg and 1.79 mmHg for the dexmedetomidine and brimonidine groups, respectively, a minimum of 42 eyes per group was required. Given that the behavior of the two human eyes in YAG laser capsulotomy is different, and in many cases, despite using the same energy and number of pulses, intraocular pressure and inflammatory changes occur quite differently in the two eyes, the second eye was considered independent and included in this study in bilateral cases.

### 2.2 Study Procedures

All patients underwent eye examination including slit‐lamp biomicroscopy and non‐dilated fundus evaluation, before enrollment. IOP and central corneal thickness (CCT) were measured using a non‐contact tonometer with integrated pachymetry (Canon TX‐20P, Tokyo, Japan). The TX‐20P measures CCT and automatically calculates a CCT‐compensated (corrected) IOP. Therefore, the IOP values reported in this study refer to the CCT‐compensated IOP provided by the device. At each time point, three consecutive measurements were obtained for each eye. The device automatically selected and displayed the best‐quality CCT‐compensated IOP reading among the three measurements, and this value was used for the final analysis. Conjunctival hyperemia was assessed at each follow‐up evaluation. The best & Corrected IOP (taking into account the effect of corneal thickness on IOP) among the three IOP measurements that selected and recorded by the tonometer device on the measurement sheet, was used in the study. To prevention of operator bias, the operator had no role in selecting the IOP. BP and heart rate were recorded, and patients were asked about potential adverse events such as dry mouth, nausea, vomiting, palpitations, or syncope. All ocular examinations and laser procedures were carried out by the same ophthalmologist.

Patients were randomly assigned dexmedetomidine 0.008% or brimonidine 0.2% eye drop treatment groups using a random number table. The eye drops were prepared daily in identical bottles labeled “A” and “B,” with the pharmacist maintaining a confidential record of their contents. Both investigators and patients were blinded to the treatment allocation. Ten minutes after administering the study drop, one drop of tropicamide 1% (Sina Darou Laboratories, Iran) was applied for pupil dilation. One hour later, Nd:YAG laser posterior capsulotomy was performed in a circular pattern 4 mm in diameter centered on the visual axis (Lightmed Corporation, LPULSA SYL9000, Taiwan) with ‘Ocular Peyman G. Capsulotomy Lens’. Laser procedural parameters were recorded for each eye, including the number of laser shots (pulses) and the total delivered laser energy (mJ). Given that post‐Nd:YAG inflammatory response and IOP elevation may be influenced by the amount of laser energy applied, these parameters were documented to reduce procedural confounding and to ensure comparability between the two randomized groups.

Although previous studies have reported mixed findings regarding the association between Nd:YAG laser parameters and post‐procedure IOP changes, we prospectively recorded the number of shots and total delivered energy for each procedure to minimize procedural confounding and to facilitate between‐group comparability.

### 2.3 Preparation of Eye Drops

Dexmedetomidine 0.008% was prepared by diluting 4 mL of dexmedetomidine‐HCl (MEDONEX 200, 0.01%, Exir, Boroujerd, Iran) in 1 mL of artificial tears (TEARLOSE, Sina Darou Laboratories, Iran) to create a 5 mL solution. This solution was stored in sterile eye drop containers. Brimonidine 0.2% (BRIMOGAN, Sina Darou Laboratories, Iran) was similarly stored in identical containers.

### 2.4 Statistical Analysis

Statistical analyses were conducted using IBM SPSS 22.0 (Inc., Chicago, IL, USA). Continuous variables were compared between the groups using independent and paired sample *t‐*tests, and reported as mean ± standard deviation (SD). For each eye and time point, the final IOP value entered into the dataset was derived from three consecutive tonometry readings as described above (device‐selected best‐quality value/mean of three readings). ANOVA and repeated measures analysis were employed to assess differences across multiple time points. A *p* value < 0.05 was considered as the threshold of statistical significance.

## 3 Results

A total of 111 eyes from 89 participants were randomly assigned to two treatment groups: dexmedetomidine (*N* = 43) and brimonidine (*N* = 46). All participants had a history of phacoemulsification surgery and significant posterior capsule opacification. Demographic data and mean intraocular pressure (IOP) levels are summarized in Table 1. The mean age of patients was 59.4 ± 15.1 years in the dexmedetomidine group and 64.2 ± 11.4 years in the brimonidine group (*p* = 0.09). The baseline mean IOP was similar in both groups: 16.3 ± 3.6 mmHg in the dexmedetomidine group and 16.7 ± 2.9 mmHg in the brimonidine group (*p* = 0.49). Procedural laser exposure was also comparable between groups. The number of shots and the total delivered laser energy did not differ significantly between the dexmedetomidine and brimonidine groups (Table 1), indicating similar Nd:YAG treatment intensity across the two arms. The mean and standard deviation of number of laser shots and total laser energy in dexmedetomidine group were 28.14 ± 4.85 and 56.21 ± 9.77 and in brimonidine group were 26.75 ± 3.82 and 55.61 ± 6.53, respectively (Mann–Whitney U test). There was no significant difference between the two groups (*p* = 0.09 and *p* = 0.71, respectively).

**TABLE 1:** Comparison of baseline characteristics between the two groups.

Variable		Dexmedetomidine	Brimonidine	*p*
Patients/eyes (*n*)		43/58	46/53	0.480
Gender (*n*, %)	Male	18 (41.9%)	18 (39.1%)	
	Female	25 (58.1%)	28 (60.9%)	
Age (mean ± SD, range)		59.4 ± 15.1 (21–87)	64.2 ± 11.4 (28–90)	0.090
PCO Severity (*n*, %)	Moderate	49 (84.5%)	38 (71.5%)	0.120
	Severe	9 (15%)	15 (28.5%)	
IOP (mean ± SD, range)	Baseline	16.3 ± 3.6 (7.5–22.4)	16.7 ± 2.9 (10.3–22.1)	0.490
	30 min	14.8 ± 4.7 (7–31.4)	13.3 ± 3.9 (7.3–24.9)	0.059
	4 h	17.1 ± 6.2 (7.6–33.9)	13.2 ± 5.50 (3.2–37)	0.001^a^
	24 h	16.7 ± 4.5 (7.4–35.1)	14.2 ± 3.9 (7.3–23.5)	0.004^a^

*Note:* IOP values represent CCT‐compensated IOP measured with the Canon TX‐20P (integrated pachymetry).

Abbreviations: IOP: intraocular pressure; N: number: PCO: posterior capsule opacity; SD: standard deviation.

^a^ Independent samples *t‐*test.

**FIGURE 1:**
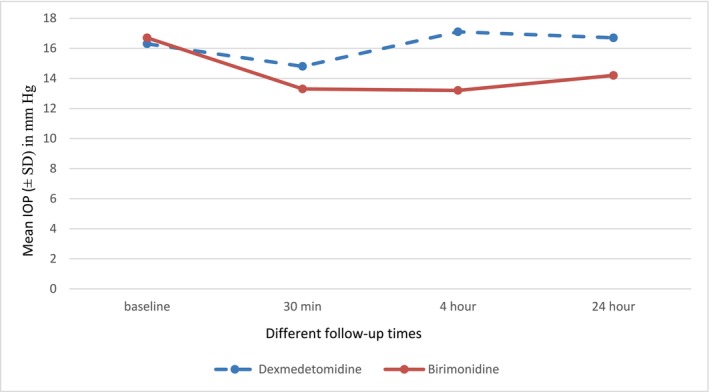
Mean IOP progression curve in dexmedetomidine and brimonidine groups across consecutive follow‐up periods.

As shown in Table 2 and Figure 1, significant differences were observed in IOP between the two groups at 4 h (*p* = 0.001) and 24 h (*p* = 0.004) after Nd:YAG laser posterior capsulotomy. In the dexmedetomidine group, the mean IOP increased to 17.1 ± 6.2 mmHg at 4 h and 16.7 ± 4.5 mmHg at 24 h, which was significantly higher than the baseline values (*p* = 0.000 for both time points). Conversely, the brimonidine group exhibited a decrease in mean IOP to 13.2 ± 5.5 mmHg at 4 h and 14.2 ± 3.9 mmHg at 24 h, which was significantly lower than baseline (*p* = 0.009 and *p* = 0.000, respectively). At 30 min post‐laser, the mean IOP in the brimonidine group was significantly lower than baseline (*p* = 0.000), while the reduction in the dexmedetomidine group was not statistically significant (*p* = 0.116).

**TABLE 2:** IOP of the same eye at different follow‐up times. All *p* values were calculated with reference to baseline IOP.

Follow‐up time	Dexmedetomidine		Brimonidine	
	IOP	*p* *	IOP	*p* *
Baseline	16.3 ± 3.6		16.7 ± 2.9	
30 min	14.8 ± 4.7	0.116	13.3 ± 3.9	< 0.001
4 h	17.1 ± 6.2	< 0.001	13.2 ± 5.50	0.009
24 h	16.7 ± 4.5	< 0.001	14.2 ± 3.9	< 0.001

Abbreviation: IOP: intraocular pressure.

* Paired samples *t‐*test.

Table 3 shows that the incidence of IOP spikes exceeding 10 mmHg at 4 h was significantly higher in the dexmedetomidine group (9%) compared with the brimonidine group (2%) (*p* = 0.035). Additionally, the incidence of IOP exceeding 30 mmHg at 4 h was marginally significant and higher in the dexmedetomidine group (7%) compared with the brimonidine group (2%) (*p* = 0.09).

**TABLE 3:** Comparison of the increase in IOP between the two groups at 4 h following laser capsulotomy.

Variable		Eyes (*N* = 111)		*p* value
		Dexmedetomidine (*N* = 58)	Brimonidine (*N* = 53)	
Increase from baseline (*n*, %)	5–10 mmHg	3 (5%)	2 (4%)	0.700
	> 10 mmHg	5 (9%)	1 (2%)	0.035*
IOP (*n*, %)	25–29 mmHg	2 (4%)	1 (2%)	0.400
	≥ 30 mmHg	4 (7%)	1 (2%)	0.090

Abbreviation: IOP: intraocular pressure.

* Chi‐square test.

### 3.1 Adverse Events

No ocular adverse events, such as hyperemia, were observed in either group. Similarly, no systemic adverse events, including hypotension, hypertension, bradycardia, tachycardia, dry mouth, nausea, vomiting, or syncope, were reported. However, as shown in Table 4, the mean systolic (*p* = 0.059) and diastolic (*p* = 0.08) blood pressure in the brimonidine group was marginally lower than that of the dexmedetomidine group, particularly at 4 h after the procedure.

**TABLE 4:** Comparison of blood pressure and pulse rate between the two groups at different follow‐up times.

Variable	Follow up time	Dexmedetomidine	Brimonidine	*p*
Systolic BP (mmHg)	Baseline	134.2 ± 20.6	133.8 ± 19.6	0.900
	30 min	131.4 ± 15.9	129 ± 16.5	0.400
	4 h	127.9 ± 18.9	120.6 ± 17.1	0.059
	24 h	134 ± 19.5	128.2 ± 15.6	0.120
Diastolic BP (mmHg)	Baseline	82.4 ± 10.8	81 ± 10.5	0.500
	30 min	80 ± 8.5	82 ± 16	0.400
	4 h	78.7 ± 9.8	75.2 ± 9.2	0.080
	24 h	79.50 ± 9.6	78.8 ± 9.8	0.500
Pulse rate (BPM)	Baseline	77 ± 12.8	80 ± 12.5	0.200
	30 min	75 ± 11.0	76 ± 13.0	0.600
	4 h	79 ± 12.0	79 ± 12.0	0.800
	24 h	78 ± 11.0	77 ± 11	0.600

Abbreviations: BP: blood pressure; BPM: beats per minute.

## 4 Discussion

PCO is a well‐known complication frequently occurring following cataract surgery, often leading to visual disturbances such as reduced acuity, contrast sensitivity, and glare [1]. Nd:YAG laser capsulotomy is an effective treatment for PCO, but it can cause an acute rise in IOP, reportedly occurring in 20%–95% of cases with increases of 5 mmHg or more [13]. This IOP elevation typically begins immediately after the procedure and peaks within 3–4 h [1, 14, 15, 16]. While this increase is usually transient, it can pose risks to vision in susceptible patients.

Brimonidine 0.2% and apraclonidine 0.5% are commonly used to prevent post‐laser IOP spikes [1]. Brimonidine, a selective α2‐adrenergic agonist, reduces IOP by mitigating the production of aqueous humor and promoting uveoscleral outflow [12]. Numerous studies have evaluated the efficacy of various anti‐glaucoma medications, including brimonidine, in alleviating IOP spikes after Nd:YAG laser capsulotomy. Dexmedetomidine, another α2‐adrenergic agonist, has shown promise in other contexts, such as peribulbar blocks during cataract and glaucoma surgeries, where it demonstrated IOP‐lowering effects comparable to intravenous administration [8, 9, 10, 11]. Additionally, studies in animals have reported IOP reductions following intramuscular dexmedetomidine injections in dogs and cats, although some studies noted IOP increases in cats [17, 18].

Topical dexmedetomidine has only recently been explored for IOP reduction. Two prior studies reported significant IOP decreases of 20%–44% in healthy human subjects using topical dexmedetomidine 0.0055% [4, 12]. To date, no study has explored the effectiveness of topical dexmedetomidine in preventing IOP spikes after Nd:YAG laser capsulotomy. Given the limited data on its use, the present work aimed to evaluate the effects of topical dexmedetomidine 0.008% and brimonidine 0.2%.

In this RCT, we compared the prophylactic effects of dexmedetomidine 0.008% and brimonidine 0.2% in 111 eyes undergoing Nd:YAG laser capsulotomy. The demographic characteristics, severity of PCO, and baseline IOP were similar between the two groups. Our findings indicate that brimonidine achieved a greater reduction in IOP compared with dexmedetomidine at all follow‐up time points. At 30 min post‐laser, the reduction in IOP was slightly higher in the brimonidine group, though the difference was not statistically significant. Nevertheless, at 4 and 24 h, brimonidine demonstrated a significantly greater reduction in IOP compared with dexmedetomidine. In the dexmedetomidine group, IOP increased above baseline levels at 4 and 24 h, while in the brimonidine group, IOP remained consistently lower than baseline throughout the follow‐up period. Despite these differences, the mean IOP in both groups remained within the normal range for human eyes. These results underscore the superior efficacy of brimonidine in maintaining lower IOP levels over time, supporting its continued role as a standard prophylactic agent in clinical practice.

The incidence of IOP spikes exceeding 10 mmHg at 4 h post‐laser was significantly higher in the dexmedetomidine group (9%) in comparison to the brimonidine group (2%). Additionally, IOP levels exceeding 30 mmHg were observed in 7% of the dexmedetomidine group versus 2% in the brimonidine group, though this difference was marginally significant. Increases in the range of 5–10 mmHg and IOP levels between 25 and 29 mmHg occurred slightly more often in the dexmedetomidine group but were not significant.

In this study, IOP was measured at 30 min to assess the early effects of a single dose of dexmedetomidine and at 4 h to evaluate its peak effect, as suggested by prior studies [12]. In one investigation involving four healthy subjects, topical dexmedetomidine 0.0055% achieved a peak IOP reduction of 40%–44% within 2–5 h, with a 20% reduction persisting at 24 h [12]. No systemic adverse events including dry mouth or sedation, were observed in drug concentrations below 0.013%. Pharmacological onset of action was noted within 1 h, with a half‐life of approximately 2–3 h and peak efficacy occurring around 5 h after administration [12].

In contrast, Fakhoury et al. found a significant IOP reduction at 30 min in the dexmedetomidine group compared with placebo (9% vs. 1.1%), but no significant reduction at 4 and 24 h [4]. In our study, the maximum IOP reduction after a single dose of dexmedetomidine was approximately 9.2%, which was not statistically significant and notably lower than the 40% reduction reported in the patent [12]. Despite using a higher concentration of dexmedetomidine than in prior studies [4, 12], the IOP‐lowering effect remained limited, suggesting that even higher concentrations or optimized formulations may be necessary to achieve stronger efficacy. Factors such as the solvent, preservatives, and pH of the solution can influence drug penetration and efficacy. For example, adding benzalkonium chloride or adjusting the solution's pH has been shown to enhance intraocular absorption [12, 19]. In our study, dexmedetomidine was dissolved in artificial tears containing 0.3% benzalkonium chloride and 0.1% dextran 70, yet the IOP reduction was less pronounced than expected.

Interestingly, IOP in the dexmedetomidine group increased by 5% and 2.5% at 4 and 24 h, respectively, compared with baseline. This contrasts with findings from earlier studies on topical dexmedetomidine [4, 12] and highlights potential limitations in its efficacy in this clinical context. Notably, this study evaluated a single dose of dexmedetomidine in patients without a history of elevated IOP or glaucoma. Its effects might be more pronounced in patients with pre‐existing elevated IOP or glaucoma [4, 20]. Furthermore, chronic use of dexmedetomidine could yield greater reductions, as most glaucoma medications require 1–2 weeks to reach full efficacy [21].

No systemic side effects were observed after the administration of a single dose of dexmedetomidine, consistent with prior studies [4, 12]. This suggests that topical dexmedetomidine is safe for short‐term use, but further research is needed to assess its long‐term safety and efficacy.

A practical limitation is that topical dexmedetomidine is not widely available as a commercial ophthalmic formulation in many settings and may require compounding, which could limit immediate routine clinical adoption despite the study's investigational value. As laser parameters, particularly total delivered energy and the number of shots, may influence the magnitude of post‐capsulotomy IOP changes, we recorded these variables for each procedure and confirmed that they were similar between the two randomized groups. This reduces the likelihood that between‐group differences in IOP were driven by procedural (laser‐related) confounding rather than the prophylactic drops.

## 5 Conclusion

In summary, topical dexmedetomidine 0.008% is a safe option with no observed systemic or ocular adverse events in this study. Both dexmedetomidine 0.008% and brimonidine 0.2%, when administered as a single dose one hour before Nd:YAG laser posterior capsulotomy, effectively prevented significant acute IOP spikes. Dexmedetomidine shows potential as a new prophylactic agent, although its IOP‐lowering effect was less pronounced compared with brimonidine.

Further research is needed to optimize its use. Specifically, studies should explore the impact of different solvents and higher concentrations, as prior studies suggest minimal side effects at concentrations below 0.013%. Additionally, investigations into its long‐term efficacy for lowering IOP in glaucoma patients are warranted. Finally, comparative studies are necessary to establish whether dexmedetomidine is non‐inferior to brimonidine in preventing post‐laser IOP spikes. These efforts will help clarify its role in clinical practice and improve treatment outcomes.

## Author Contributions

H.O.: designed the study; H.O. and P.Z.: conducted the study; G.A., H.O., and P.Z.: wrote the paper; F.A. performed the statistical analysis of the study; H.O.: has primary responsibility for the final content; and all authors have read and approved the final manuscript.

## Funding

The authors have nothing to report.

## Disclosure

The authors have nothing to report.

## Ethics Statement

The study and consent procedure were approved by the Ardabil University of Medical Sciences Ethics Committee (No: IR.ARUMS. REC.1402.148) with IRCT NO (20 231 008 059 648 N1) and followed the tenets of the Declaration of Helsinki.

## Consent

All patients signed consent to participate and consent to publish.

## Conflicts of Interest

The authors declare there is no conflicts of interest.

## Data Availability

The data that supports the findings of this study are available if requested.
